# Heterozygous *TREX1* variants in a neonate with Aicardi-Goutières syndrome type 1: a case report

**DOI:** 10.1515/biol-2025-1391

**Published:** 2026-11-02

**Authors:** Jie Luo, Xiaojiao Wang, Ying Liu, Jingwen Weng, Huanyu Luo, Mingyan Hei

**Affiliations:** Department of Neonatology, Neonatal Center, Beijing Children’s Hospital, Capital Medical University, Beijing, 100045, China; Department of Radiology, Beijing Children’s Hospital, Capital Medical University, National Center for Children’s Health, Beijing, 100045, China

**Keywords:** Aicardi-Goutières syndrome, *TREX1*, ruxolitinib, whole exome sequencing, mutation

## Abstract

Aicardi-Goutières syndrome type 1 (AGS1) is a rare autosomal recessive inflammatory disorder caused by mutations in *TREX1* gene, and is frequently misdiagnosed as congenital or neonatal central nervous system infection because of its overlapping clinical manifestations. We report a 27-day-old male neonate who presented with recurrent fever, a persistent cerebrospinal fluid (CSF) pleocytosis, and poor response to broad-spectrum antimicrobial therapy. Imaging revealed diffuse white matter abnormalities and bilateral basal ganglia calcifications. Extensive microbiological investigations were negative, prompting genetic testing. Whole exome sequencing identified two compound heterozygous variants in *TREX1*: c.294dup (p.Cys99MetfsTer3) and c.868_885del (p.Pro290_Ala295del), inherited from his mother and father, respectively. Based on the clinical manifestations, neuroimaging findings, and genetic results, a diagnosis of AGS1 was established. Treatment with the Janus kinase inhibitor ruxolitinib reduced the frequency and severity of febrile episodes. However, the patient developed microcephaly, neurodevelopmental delay, and motor impairment in infancy. We reported a rare case of AGS1 associated with two compound heterozygous variants, including c.294dup (p.Cys99MetfsTer3) and c.868_885del (p.Pro290_Ala295del). This case expands the genotypic and phenotypic spectrum of *TREX1*-associated AGS1 and highlights the importance of considering monogenic interferonopathies in neonates presenting with sterile CSF inflammation, intracranial calcifications, and treatment-refractory fever.

## 1 Introduction

Aicardi-Goutières syndrome (AGS) is a rare inherited interferonopathy characterized by severe neuroinflammation and immune dysregulation, primarily affecting the central nervous system (CNS) [1]. The disorder typically manifests during infancy and is characterized by early-onset encephalopathy, intracranial calcifications, white matter abnormalities, microcephaly, and varying degrees of neurological impairment [2]. To date, pathogenic variants in 9 genes, including *TREX1*, *RNASE2A/B/C*, *SAMHD1*, *ADAR1*, *IF1H1*, *LSM11*, and *RNU7-1* [3].

Among the AGS subtypes, AGS type 1 (AGS1, OMIM: 225750) is caused by biallelic pathogenic variants in *TREX1* and is generally considered the most severe form, often presenting during the neonatal period [3], 4]. *TREX1* encodes a 3′–5′ DNA exonuclease responsible for degrading endogenous DNA [5]. Loss of *TREX1* function results in the accumulation of intracellular nucleic acids, leading to inappropriate activation of the type I interferon (IFN-I) pathway and chronic inflammation. Consequently, affected individuals develop a spectrum of autoimmune, inflammatory, and neurodegenerative manifestations [6], 7].

The diagnosis of AGS1 remains challenging, particularly in neonates, because its initial manifestations often mimic congenital infections or neonatal encephalitis. Patients frequently present with fever, cerebrospinal fluid pleocytosis, intracranial calcifications, and white matter abnormalities in the absence of identifiable pathogens [8], 9]. Given the marked clinical heterogeneity and rarity of the disease, genetic testing, particularly whole-exome sequencing (WES), has become an essential diagnostic tool for establishing an early and accurate diagnosis.

Here, we report a Chinese neonate with AGS1 who presented with recurrent fever, persistent sterile cerebrospinal fluid inflammation, and characteristic neuroimaging abnormalities. WES identified compound heterozygous *TREX1* variants, c.294dup (p.Cys99MetfsTer3) and c.868_885del (p.Pro290_Ala295del), inherited from his mother and father, respectively. This case expands the genotypic and phenotypic spectrum of *TREX1*-associated AGS1 and highlights the importance of considering monogenic interferonopathies in neonates with unexplained neuroinflammatory manifestations.

## 2 Case presentation

A 27-day-old male neonate, born at 38 weeks of gestation, was admitted to our department because of fever and subtle hypo-reactivity to stimulation. His maximum body temperature during the first 3 days after admission was 38.8 °C. He had a birth weight of 2,760 g and APGAR scores of 10 at both 1, 5 and 10 min after birth. Both parents are healthy His family history was notable for healthy parents, a healthy elder sister and a pervious pregnancy that had been terminated at 26 weeks of gestation because of severe cardiovascular dysplasia.

On admission, physical examination showed mildly reduced mental response and along with slightly anemic appearance. Cardiopulmonary and abdominal examinations were generally normal. Muscle tone was normal in all limbs, and primitive reflexes were automatically elicited.

Laboratory investigations showed a normal peripheral white blood cell (WBC) count (5.16–9.50 × 10^9^/L), and mildly decreased hemoglobin levels (82–102 g/L). Cerebrospinal fluid (CSF) analysis revealed pleocytosis, with WBC count ranging from 13 to 56 × 10^6^/L and mononuclear cells of 86–96 % of total cells. CSF glucose concentration of 2.39–3.49 mmol/L, while CSF protein levels remained within the normal range. Extensive infectious investigations, including blood culture, TORCH serology, and high-throughput metagenomic sequencing of CSF, failed to identify any pathogenic microorganisms. Liver, renal, and thyroid function tests were normal. Urine screening revealed a slight elevation in glutaric acid, along with increased free carnitine to palmitoylcarnitine (C0/C16) and butyrylcarnitine to propionylcarnitine (C4/C3) ratios.

Ophthalmologic examination revealed subretinal hemorrhage in the left eye, whereas hearing screening was normal. Echocardiography and abdominal ultrasound showed no abnormalities. Cranial ultrasonography revealed bilateral subependymal hyperechoic lesions, measuring approximately 0.7 × 0.6 × 1.9 cm on the left and 0.9 × 0.7 × 2.0 cm on the right, consistent with bilateral subependymal hemorrhage in the resorption stage. Brain magnetic resonance imaging (MRI) with contrast revealed scattered punctate and linear subependymal T1-weighted hyperintense foci along the bilateral lateral ventricles, a thin cerebral cortex and T2-weighted imaging hyperintensities within white matter without meningeal enhancement (Figure 1). Cranial computer tomography (CT) revealed multiple bilateral symmetrical subcortical hypodense lesions (mean attenuation, approximately 11 HU), scattered punctate and linear hyperdense foci within the bilateral cerebral cortex, and symmetric punctate-to-linear calcifications in the bilateral basal ganglia A mega cisterna magna was present. No ventricular dilatation, midline shift, or cerebellar abnormality was observed (Figure 2).

**Figure 1:**
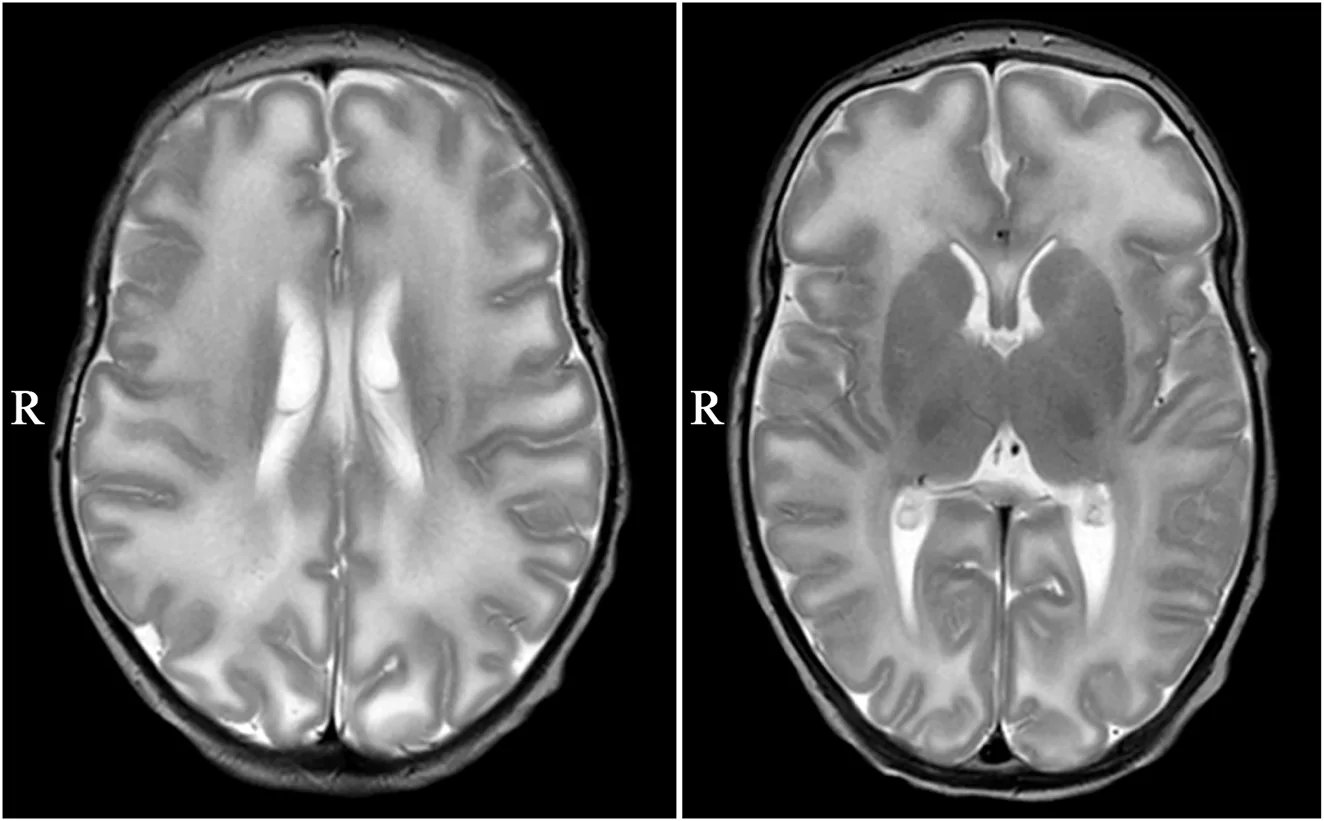
Brain MRI of proband. Brain MRI revealed scattered punctate and linear subependymal T1-weighted hyperintense foci along the bilateral lateral ventricles, a thin cerebral cortex and T2-weighted imaging hyperintensities within white matter without meningeal enhancement.

**Figure 2:**
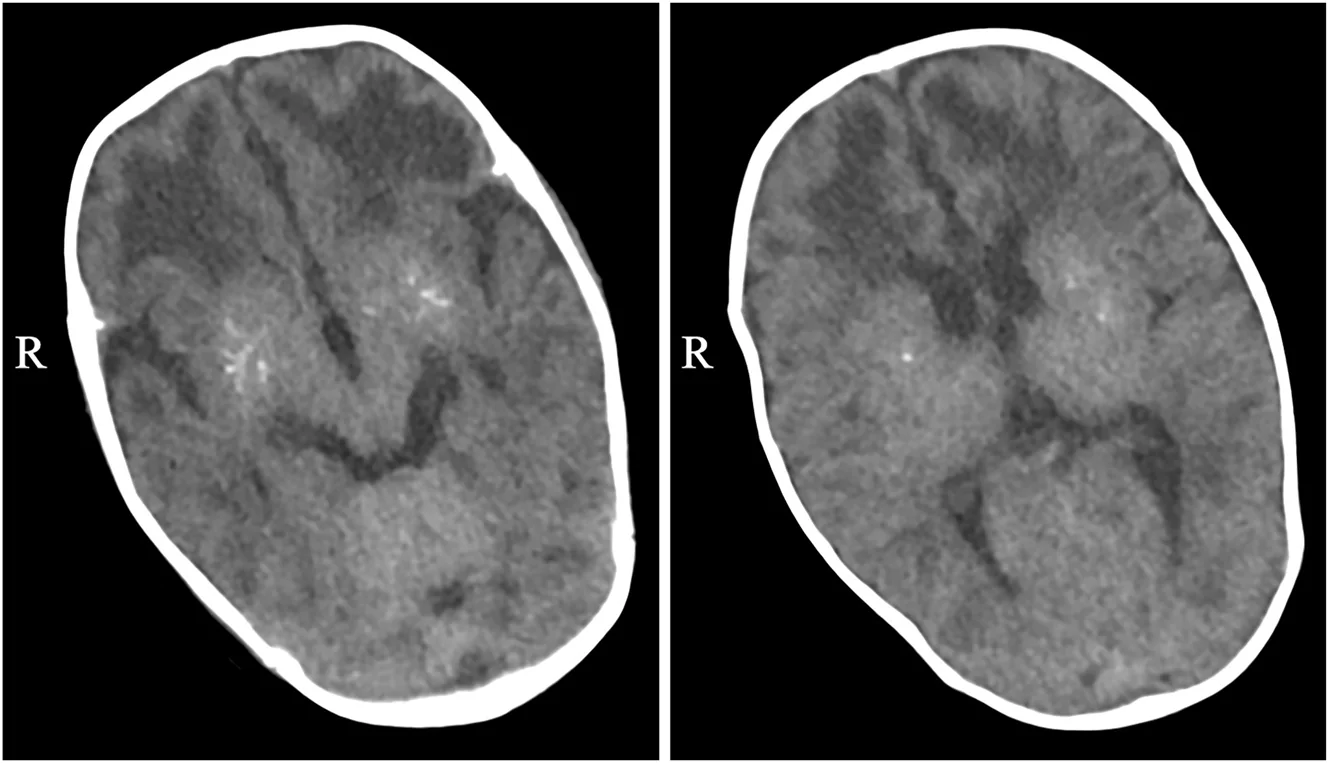
Cranial CT findings of the proband. Cranial CT revealed multiple bilateral symmetric subcortical hypodense lesions (mean attenuation, approximately 11 HU), scattered punctate and linear hyperdense foci within the bilateral cerebral cortex, and symmetric punctate-to-linear calcifications in the bilateral basal ganglia A mega cisterna magna was present. No ventricular dilatation, midline shift, or cerebellar abnormality was observed.

Given the initial suspicion of CNS infection, empirical antimicrobial with ceftazidime and penicillin was initiated on hospital day 13 and continued until hospital day 21. However, on hospital day 24, the patient developed recurrent fever with a peak temperature of 39.2 °C, prompting the re-initiation of antimicrobial therapy. Treatment was discontinued again on hospital day 30 because repeated microbiological investigations remained negative. Throughout hospitalization, the patient experienced recurrent febrile episodes, and CSF pleocytosis persisted despite broad-spectrum antimicrobial treatment. Repeated microbiological testing, including CSF pathogen detection, failed to identify an infectious agent, making an infectious etiology unlikely.

Considering the neonatal onset, persistent inflammatory manifestations, characteristic neuroimaging abnormalities, and poor response to antimicrobial therapy, an inherited autoinflammatory disorder was suspected. WES was therefore performed to identify potential pathogenic variants associated with hereditary inflammatory or neurological diseases.

After obtaining informed consent, peripheral blood samples from the patient and his parents, and additional family members for genetic analysis. Genomic DNA was extracted using the QIAamp DNA Blood Kit (Qiagen). WES was performed by MyGenostics Inc. (Beijing, China) using GenCap technology to capture coding exons and flanking regions. Paired-end sequencing (150 bp reads) was conducted on the Illumina NextSeq 500 platform. After quality control, the remaining sequences were aligned to the human reference genome (hg19) using BWA software. Single nucleotide polymorphisms (SNPs) and insertions/deletions (indels) were called using GATK, and their pathogenicity was predicted using SIFT, PolyPhen2, MutationTaster, and GERP++. Variants with a frequency of <0.05 predicted to be pathogenic in public and MyGenostics internal databases were considered potentially disease-related. Candidate variants were subsequently validated by Sanger sequencing following PCR amplification, using the ABI 3730xl sequencer (Applied Biosystems, USA).

The exon structure and protein domains of the *TREX1* gene are shown in Figure 3A. WES identified two heterozygous *TREX1* variants in the proband: c.294dup (p.Cys99MetfsTer3) (PVS1, PM3_VeryStrong) and c.868_885del (p.Pro290_Ala295del) (PM3_VeryStrong, PM4), both of which were confirmed by Sanger sequencing. The c.868_885del (p.Pro290_Ala295del) variant was inherited from the father, whereas t the c.294dup (p.Cys99MetfsTer3) variant was inherited from the mother (Figure 3B and C). Both variants are rare in population databases (allele frequencies: 0.2567 % for c.294dup and 0.0066 % for c.868_885del), supporting their rarity in the general population. According to the American College of Medical Genetics and Genomics (ACMG) guidelines, c.294dup (p.Cys99MetfsTer3) was classified as pathogenic, whereas the c.868_885del (p.Pro290_Ala295del) was classified as likely pathogenic (Table 1).

**Figure 3:**
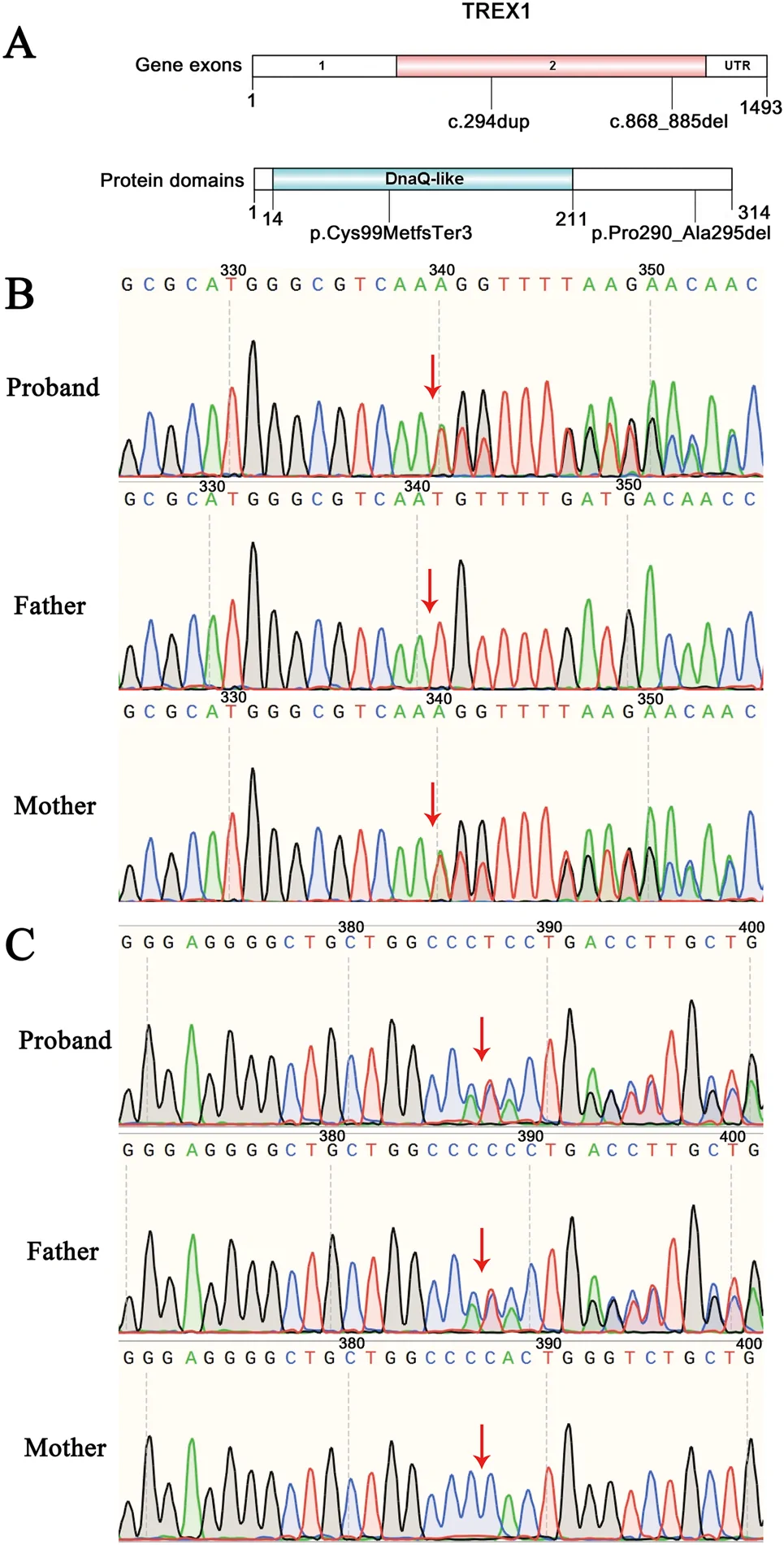
*TREX1* gene and its variant spectrum identified in the proband. (A) Schematic representation of the exon structure and protein domains of *TREX1*. (B) Whole-exome sequencing (WES) identified the heterozygous c.294dup (p.Cys99MetfsTer3) variant in the proband, which was inherited from his mother. (C) WES identified the heterozygous c.868_885del (p.Pro290_Ala295del) variant in the proband, which was inherited from his father.

**Table 1:** The pathogenicity of *TREX1* variants.

Gene	Chromosomal location	Transcript	Exon	Nucleotide change	Amino acid change	Variant type	ACMG classification	ACMG evidence	Bayesian score	Population database frequency
*TREX1*	chr3:48508348-48508349	NM_033629.6	Exon2	c.294dup	p.Cys99MetfsTer3	Heterozygous	Pathogenic	–	–	0.0025674
	chr3:48508922-48508939			c.868_885del	p.Pro290_Ala295del	Heterozygous	Likely pathogenic	PM2_Supporting, PM3, PM4	5	0.0000663

Extended family screening revealed that the paternal grandmother, paternal aunt, paternal uncle, and female cousin carried the heterozygous c.868_885del (p.Pro290_Ala295del) variant, whereas the proband’s sister and maternal grandfather carried the heterozygous c.294dup (p.Cys99MetfsTer3) variant (Figure 4).

**Figure 4:**
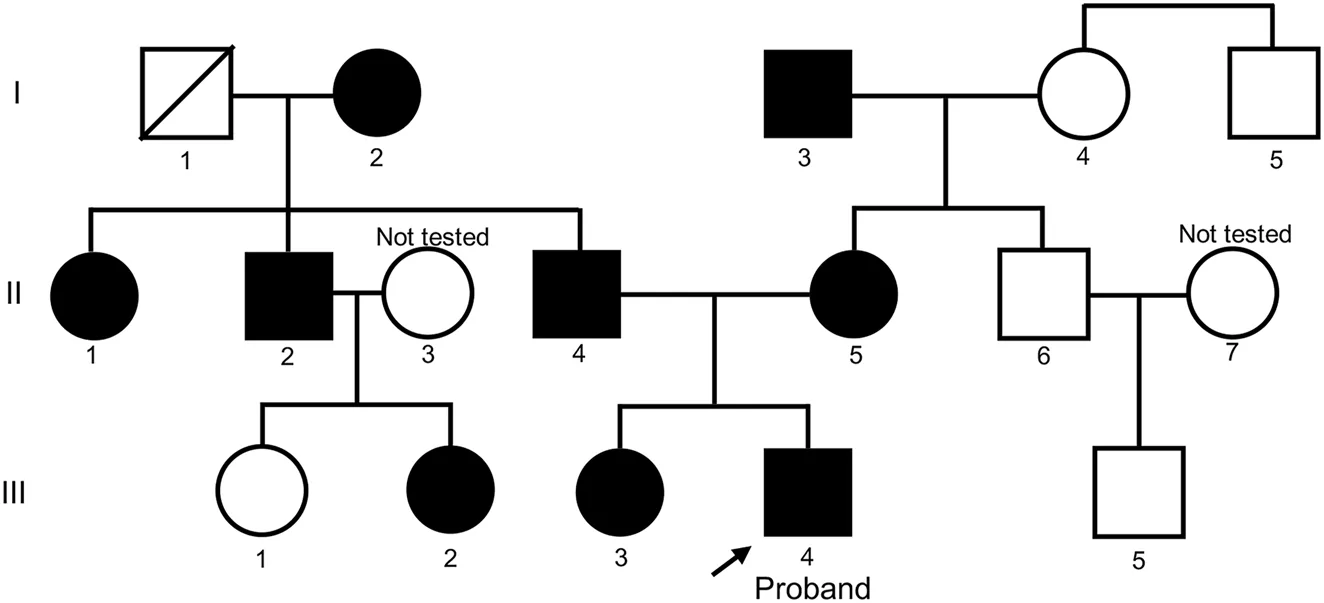
Pedigree analysis of the family with *TREX1* variants. The proband’s paternal grandmother, aunt, uncle, and female cousin carried the c.868_885del (p.Pro290_Ala295del) heterozygous variant. The proband’s sister and maternal grandfather carried the c.294dup (p.Cys99MetfsTer3) heterozygous variant. The inheritance pattern of the identified *TREX1* variants is consistent with compound heterozygosity in the proband.

To investigate the potential structural consequences of the two variants, three-dimensional models were generated using SWISS-MODEL database (https://swissmodel.expasy.org/) and visualized with PyMOL. Structural analysis showed that the c.294dup (p.Cys99MetfsTer3) variant led to the substitution of cysteine (Cys) at position 99 with methionine (Met) (Supplementary Figure 1A and B). The c.868_885del (p.Pro290_Ala295del) variant resulted in the deletion of amino acids 290–295 (Supplementary Figure 1C and D).

Based on clinical manifestations, neuroimaging findings, and genetic results, the patient was diagnosed with AGS1. For the treatment, Janus kinase (JAK) inhibitor ruxolitinib was administered at a dose of 7.5 mg/day and continued 3 months. During treatment, febrile episodes became less frequent, decreasing from approximately once per week to once every two weeks, and the peak body temperature decreased to 37.5 °C.

At 4 months of the age, only occasional low-grade fevers were observed. However, the patient developed somnolence and exhibited a positive cortical thumb sign. He remained unable to roll over without assistance, and showed increased muscle tone.

At the 18-month follow-up, intermittent low-grade fever persisted. His height and weight were approximately at the 25th percentile according to Chinese male pediatric growth chart. He had erupted five teeth and showed no chilblain-like rash. Severe developmental delay was evident, as he was unable to maintain head control, sit, roll over, or stand. Nevertheless, he retained visual tracking ability and responded with smiling toward familiar caregivers. His diet consisted primarily of pureed carbohydrates and fruits, supplemented with formula milk. Swallowing and bowel functions remained largely preserved.

The reporting of this study conforms to CARE guidelines [10].

**Informed consent:** Informed consent was obtained from all individuals participating in this study, or from their legal guardians or wards.

**Ethical approval:** The research related to human use has been complied with all the relevant national regulations, institutional policies and in accordance with the tenets of the Helsinki Declaration, and has been approved by the Ethical Committee of Beijing Children’s Hospital, Capital Medical University (approval No.: 2025-E-093-R).

## 3 Discussion

Among the known AGS-related genes, *TREX1* plays a critical role in maintaining intracellular nucleic acid homeostasis by degrading abnormal DNA and preventing inappropriate immune activation [11]. Pathogenic variants in *TREX1* result in the accumulation of endogenous nucleic acids, activating type I IFN-mediated immune response that contributes to the pathogenesis of AGS1 [12], [13], [14]. In this report, we described a neonate case with *TREX1*-related AGS1 harboring two compound heterozygous variants, c.294dup (p.Cys99MetfsTer3) and c.868_885del (p.Pro290_Ala295del). The novelty of this case lies in its early neonatal presentation with persistent inflammatory manifestations mimicking CNS infection, highlighting the diagnostic challenges of *TREX1*-related disorders and expanding the early-onset phenotypic spectrum of AGS1.

The diagnosis of AGS can be challenging because its clinical manifestations overlap with various neurological, infectious and autoimmune disorders. In the present case, the patient exhibited normal cardiopulmonary and abdominal examinations, normal muscle tone, and preserved neonatal reflexes. However, the presence of recurrent fever, CSF inflammation, slightly elevated glutaric acid, increased C0/C16 and C4/C3 ratios, and abnormal neuroimaging findings promoted consideration of potential differential diagnosis, particularly CNS infection and autoimmune disorders. Although CSF leukocytosis was observed, the increased cells were mainly monocytes, while CSF glucose and protein levels were within normal ranges. In addition, bacterial culture, fungal culture, CSF pathogen nucleic acid testing, and TORCH-IgM tests were all negative. No obvious signs of meningeal and spinal cord were observed on contrast-enhanced MRI, making CNS infection unlikely. Considering the early onset and inflammatory features, autoimmune disorders were also considered. However, the patient had no family history of autoimmune diseases and did not exhibit characteristic symptoms such as anhidrosis, dry skin, recurrent diarrhea, oral ulcers, rashes, or feeding difficulties at the initial stage. Immunological assessments, including lymphocyte subset analysis, immunoglobulin profiling, and autoantibody screening, showed normal results, ruling out primary immunodeficiency and autoimmune diseases such as SLE. Eventually, the persistence of unexplained inflammatory features combined with characteristic neuroimaging abnormalities prompted to genetic testing, confirming the diagnosis of *TREX1*-related AGS.

The *TREX1* gene, located on chromosome 3p21.31, encodes the three-prime repair exonuclease 1 protein. *TREX1* mutations lead to the accumulation of self-DNA in the cytosol of *TREX1*-deficient cells, further triggers systemic inflammation and uncontrolled autoimmunity by chronic activation of checkpoint signaling and GMP-AMP synthase (cGAS)–stimulator of interferon genes (STING)-mediated type I interferon response [5], 13], 15]. Disruption of innate immune regulation is a central pathogenic mechanism in AGS, and disrupted *TREX1* enzyme fails to maintain host innate immune tolerance to cytosolic self-DNA and results in an abnormal innate immune response. In this study, WES identified two heterozygous variants in *TREX1*, c.294dup (p.Cys99MetfsTer3) and c.868_885del (p.Pro290_Ala295del), inherited from the mother and father, respectively. To date, only a limited number of cases involving two variants have been reported. Ji et al. described a case of a 6-year-7-month-old male patient with AGS carrying these variants, who exhibited severe developmental delay, microcephaly, and brain MRI findings similar to those observed in the present case. However, unlike the present case, the affected child also presented with limb torsion and chilblain-like lesions on the extremities [16]. Additionally, Zheng et al. reported a case carrying the heterozygous c.294dup variant in *TREX1*. The variant results in a frameshift mutation and premature termination of the protein-coding sequence [17]. Gowda et al. have reported the c.868_885del variant in the dileucine-repeat region of the *TREX1* protein [18]. The c.868_885del (p.Pro290_Ala295del) variant results in an in-frame deletion of six amino acids within the exonuclease domain, which may disrupt the structural integrity and enzymatic function of *TREX1*. Structural modeling further suggested significant alternation in the mutant protein, which may affect its exonuclease activity and leading to nucleic acid accumulation, thereby activating the innate immune response. Familial analysis in this study further supports that AGS1 is primarily caused by loss-of-function variants in *TREX1*, as heterozygous carriers within the family remained asymptomatic, consistent with the autosomal recessive pattern of inheritance. Furthermore, this study highlights the importance of genetic screening for the early diagnosis of AGS1, providing valuable insights into the clinical management of similar cases.

JAK inhibitors have emerged as a promising therapeutic agent for AGS by targeting the JAK-signal transducer and activator of transcription (STAT) pathway, which mediates IFN signaling [19]. In this case, treatment with ruxolitinib, a selective JAK1/2 inhibitor, resulting in improvement in febrile episodes, with an increased interval between episodes and a reduction in peak temperature. These findings are consistent with prior studies describing the efficacy of JAK inhibitors (e.g. baricitinib, ruxolitinib, tofacitinib) in AGS [4], [20], [21], [22]. Despite these positive outcomes, the patient continued to show neurodevelopmental impairment, including microcephaly, poor auditory and visual tracking, and delayed motor and cognitive milestones. These persistent neurological deficits highlight the challenges in reversing established neuroinflammatory damage in AGS, emphasizing the importance of early intervention. Long-term follow-up and larger cohort studies are required to further evaluate the safety and efficacy of JAK inhibition in AGS.

Several diagnostic limitations should be acknowledged. First, the early clinical manifestations of AGS1, including recurrent fever, cerebrospinal fluid inflammation, and neurological abnormalities, may mimic infectious diseases, which can delay recognition of this rare genetic disorder. Second, although whole-exome sequencing identified two heterozygous *TREX1* variants consistent with the clinical phenotype, functional validation of their effects on *TREX1* activity and downstream interferon signaling was not performed. Therefore, the diagnosis was mainly supported by clinical manifestations, neuroimaging findings, and genetic evidence. Early recognition and genetic testing remain essential for timely diagnosis and individualized management of AGS1.

## 4 Conclusions

We reported a neonatal case of *TREX1*-related AGS1 caused by two compound heterozygous variants, c.294dup (p.Cys99MetfsTer3) and c.868_885del (p.Pro290_Ala295del), which expanded the early-onset phenotypic spectrum of *TREX1*-associated diseases. This case emphasizes the need to consider genetic autoinflammatory disorders in infants with persistent inflammation and neurological abnormalities. The patient was treated with the JAK inhibitor ruxolitinib, leading to a reduction in febrile episodes but persistent neurodevelopmental impairment. These findings support the potential of JAK inhibition in managing AGS-related inflammation but highlight the need for further research to optimize treatment strategies and improve neurological outcomes in affected patients.

## Supplementary Material

Supplementary Material
