# Supplementary material for: Heterozygous TREX1 variants in a neonate with Aicardi-Goutières syndrome type 1: a case report
Source: Open Life Sci. 2026 Nov 2;21(1):20251391. doi: 10.1515/biol-2025-1391 (PMC13725325; doi:10.1515/biol-2025-1391)
Supplement: Supplementary file 1 [file j_biol-2025-1391_suppl_001.pdf]

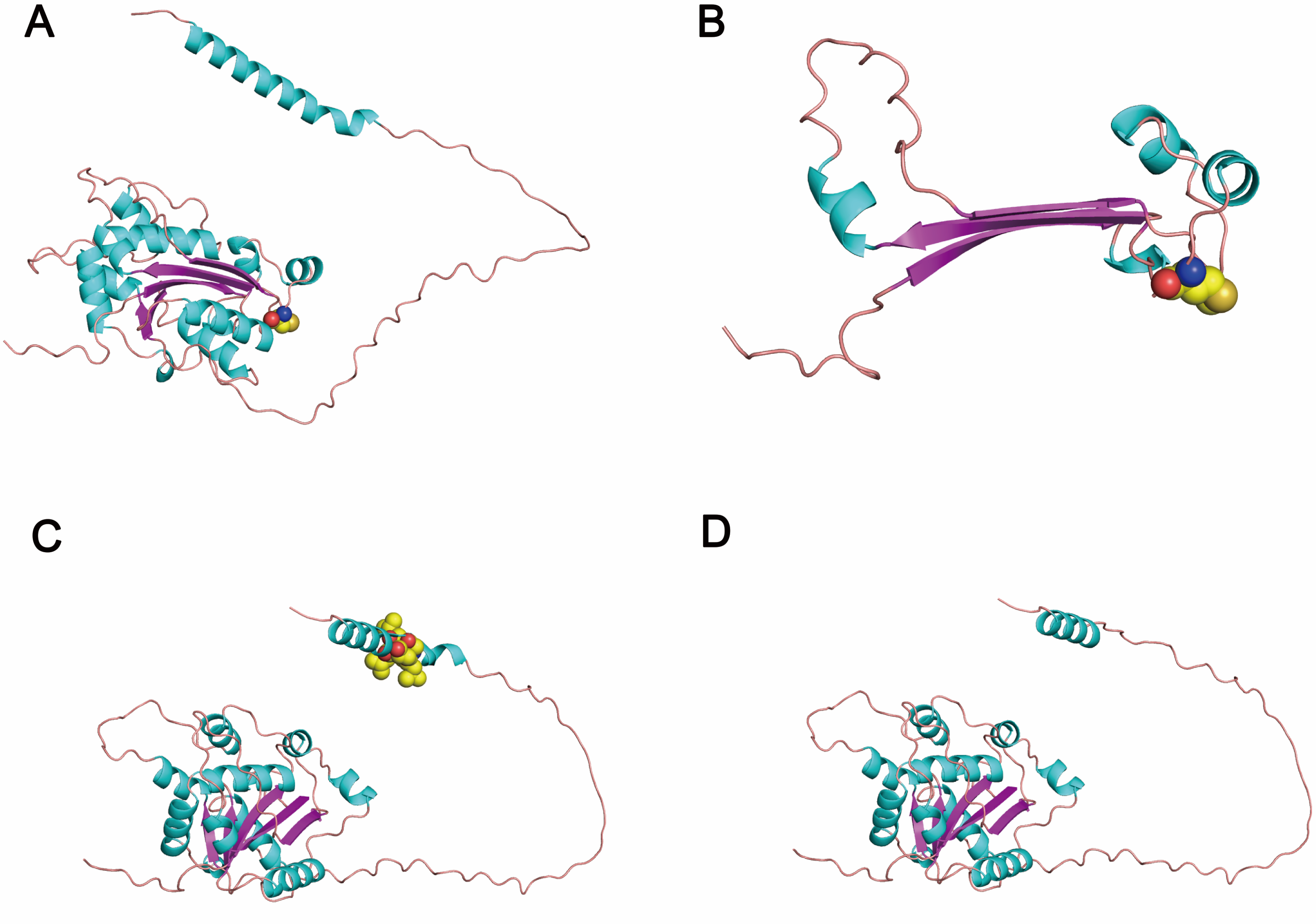

Supplementary Figure 1. Structures of wild-type and mutant TREX1. (A, B) The c.294dup (p.Cys99MetfsTer3) variant results in the substitution of cysteine (Cys) at position 99 with methionine (Met). (C, D) The c.868_885del (p.Pro290_Ala295del) variant leads to the deletion of amino acids at positions 290-295. Cyan represents α-helices, purple represents β-sheets, and pink coils represent loop structures.
